# Design and Characterization of a Dual-Protein Strategy for an Early-Stage Assay of Ovarian Cancer Biomarker Lysophosphatidic Acid

**DOI:** 10.3390/bios14060287

**Published:** 2024-06-02

**Authors:** Katharina Davoudian, Sandro Spagnolo, Navina Lotay, Monika Satkauskas, Gábor Mészáros, Tibor Hianik, Zsófia Keresztes, Gilbert Walker, Michael Thompson

**Affiliations:** 1 Department of Chemistry, University of Toronto, 80 St. George Street, Toronto, ON M5S 3H6, Canada; k.davoudian@mail.utoronto.ca (K.D.); navina.lotay@mail.utoronto.ca (N.L.); m.satkauskas@mail.utoronto.ca (M.S.); gilbert.walker@utoronto.ca (G.W.); 2 Faculty of Mathematics, Physics and Informatics, Comenius University, Mlynská dolina F1, 842 48 Bratislava, Slovakia; spagnolo2@uniba.sk (S.S.); tibor.hianik@fmph.uniba.sk (T.H.); 3 Functional Interfaces Research Group, Institute of Materials and Environmental Chemistry, HUN-REN Research Centre for Natural Sciences, Magyar tudósok krt. 2., H-1117 Budapest, Hungary; meszaros.gabor@ttk.hu (G.M.); keresztes.zsofia@ttk.hu (Z.K.)

**Keywords:** lysophosphatidic acid, gelsolin, actin, ovarian cancer, antifouling linker, EMPAS, chemiluminescence, nanoparticles, atomic force microscopy

## Abstract

The overall 5-year survival rate of ovarian cancer (OC) is generally low as the disease is often diagnosed at an advanced stage of progression. To save lives, OC must be identified in its early stages when treatment is most effective. Early-stage OC causes the upregulation of lysophosphatidic acid (LPA), making the molecule a promising biomarker for early-stage detection. An LPA assay can additionally stage the disease since LPA levels increase with OC progression. This work presents two methods that demonstrate the prospective application for detecting LPA: the electromagnetic piezoelectric acoustic sensor (EMPAS) and a chemiluminescence-based iron oxide nanoparticle (IONP) approach. Both methods incorporate the protein complex gelsolin–actin, which enables testing for detection of the biomarker as the binding of LPA to the complex results in the separation of gelsolin from actin. The EMPAS was characterized with contact angle goniometry and atomic force microscopy, while gelsolin–actin-functionalized IONPs were characterized with transmission electron microscopy and Fourier transform infrared spectroscopy. In addition to characterization, LPA detection was demonstrated as a proof-of-concept in Milli-Q water, buffer, or human serum, highlighting various LPA assays that can be developed for the early-stage detection of OC.

## 1. Introduction

Ovarian cancer (OC) patients are commonly afflicted with vague or limited symptoms rendering early detection a significant challenge in the absence of a genuinely reliable screening method. The disease is the most fatal gynecological cancer because it is usually detected at a late stage when the 5-year survival rate plummets from above 90% (stage I) to less than 30% (stage IV) [1,2]. The only available OC test is unreliable as it detects the cancer antigen 125 (CA-125) biomarker, which is elevated in about half of early-stage patients. Other strategies require imaging techniques such as the transvaginal ultrasound technique that are not suitable for rapid and low-cost detection [3]. Therefore, developing a biosensor for detecting early-stage OC is crucial to decrease mortality rates. 

Designing an OC screening test requires biomarkers that correlate with early-stage disease progression. Around 90% of stage I patients have higher levels of the bioactive phospholipid lysophosphatidic acid (LPA) [4,5], a biomarker that can identify OC since average healthy levels of LPA are 1.2 µM, while OC can cause LPA concentrations of 1.3 to 50 µM [4,5]. An LPA screening test can potentially identify and monitor the progression of the disease since the level of LPA increases with OC development [3,4,5,6]. One approach for detecting LPA utilizes the gelsolin(1-3)–actin protein complex [6], which is separated upon LPA binding. Molecular dynamics simulations of the LPA/gelsolin(1-3)–actin complex indicate that LPA’s hydrophobic tail binds through a tunnel-like region in actin, the LPA-insertion pocket, while the polar headgroup of LPA interacts with gelsolin(1-3)’s PIP_2_-binding domain [7]. As a result, gelsolin(1-3)–actin is a promising probe for the detection of a small molecule like LPA.

The small size of LPA makes it challenging to detect; however, its interaction with the gelsolin(1-3)–actin complex can be employed to enhance sensor sensitivity. We applied the protein complex to acoustic and chemiluminescent methods and assessed if these techniques were suitable for detecting LPAs. The acoustic technique utilizes the electromagnetic piezoelectric acoustic sensor (EMPAS) and detects LPAs via a change in resonance. The chemiluminescence approach incorporates iron oxide nanoparticles (IONPs) and measures LPA through the concentration of chemiluminescent dye. 

The EMPAS is an interface-sensitive device based on a piezoelectric quartz crystal, where the change in resonance frequency of the device correlates with material adsorbed on the surface. As the EMPAS quartz discs are electrodeless, surface chemistry for functionalization is ideally achieved using the silanization approach. Ultra-high acoustic frequencies are applied in the EMPAS, making the device far more sensitive compared to the conventional thickness-shear mode sensors [8]. The gelsolin(1-3)–actin complex can be immobilized on the surface of a quartz disc. The dissociation of actin from the protein complex due to the presence of LPA results in a decrease in sensing layer material and hence an increase in EMPAS resonance frequency, which serves as an analytical signal for LPA detection.

Our other LPA assay combines the sensitivity of chemiluminescence with the versatility of nanoparticles. Nanoparticles, in particular IONPs, can be used as a platform for the gelsolin(1-3)–actin complex that does not involve a stationary surface or the flow of reagent over a surface. IONPs are superparamagnetic and can therefore be easily and quickly separated from a solution with a magnet. The IONPs are functionalized with gelsolin(1-3)–actin, where actin is tagged with a chemiluminescent dye. After incubating and separating the IONPs with a sample, the remaining sample can then be analyzed for the presence of the dye. The dye should only come from the displacement of actin by LPA, and therefore dye concentration directly correlates with the presence of LPA in the sample. 

We have previously shown the success of a similar system that used fluorescence [6]. The switch to chemiluminescence was particularly motivated by the high sensitivity of the technique. Our previous fluorescence system had a detection limit of 5 μM, but as stated above, clinically relevant assays need to be able to detect sub-1 μM concentrations of LPA. Chemiluminescence is approximately 10^3^ times more sensitive than fluorimetry, with detection limits as low as 0.1 fmol documented [9]. This sensitivity is achievable through the fast reaction kinetics that cause chemiluminescence and the absence of incident light, making it a true dark-background experiment and eliminating considerations such as scattering, background incident signal, or issues caused by the instability of the light source [9]. This enhanced sensitivity should help to achieve an accurate detection of clinically relevant LPA concentrations.

**Figure 1:**
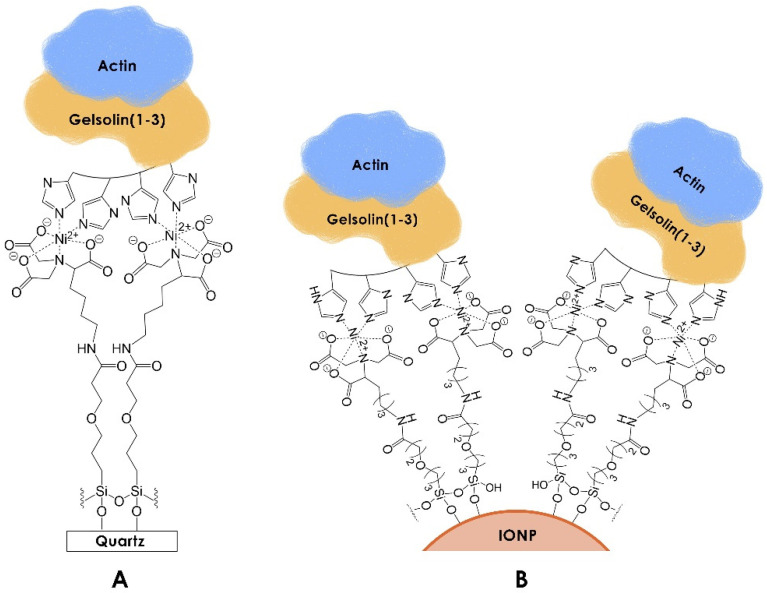
Functionalized (**A**) EMPAS discs and (**B**) iron oxide nanoparticles for detecting LPA via gelsolin(1-3)–actin. Although recombinant gelsolin(1-3) has an 8-histidine tag, four are illustrated for clarity. The illustration is not to scale.

To enable sensing in complex matrices like a serum, both the acoustic and chemiluminescent sensing methods incorporated antifouling layers. This is a highly essential property that enables sensitive and specific detection in complex biological samples by limiting non-specific adsorption (NSA) [10]. Furthermore, the antifouling molecules used in these measurements also function as tandem linkers (Figure 1). The silane antifouling linker 3-(3-(trichlorosilyl)propoxy)propanoyl chloride (MEG-Cl) [11] was applied to both the EMPAS and the IONPs, enabling a linkage to the gelsolin(1-3)–actin complex via nickel–nitrilotriacetic acid (Ni-NTA). The linker possesses polar functional groups that can facilitate interactions with water molecules, promoting the formation of a water barrier. These structured water molecules render NSA unfavourable, allowing for rapid detection in complex samples without extensive and time-consuming preprocessing [10,12]. 

This study presents the characterization of the acoustic and chemiluminescent sensors and the proof-of-concept detection of LPA. Contact angle goniometry and atomic force microscopy characterized the acoustic sensor, while transmission electron microscopy and Fourier transform infrared spectroscopy characterized the chemiluminescent nanoparticle approach. While the functionalization of the sensing platforms was successful, further work is needed to establish the potential detection of LPA with the acoustic and chemiluminescent sensors. 

## 2. Materials and Methods

### 2.1. Materials

All materials were purchased from Sigma-Aldrich (Oakville, ON, Canada) unless noted otherwise. Published procedures were followed for the synthesis of MEG-Cl [11] and gelsolin protein expression [6]. His-tagged recombinant gelsolin(1-3) was dissolved in HEPES buffer (20 mM HEPES, 1 mM CaCl_2_, pH 7.4). Actin was solubilized in a 1:3 solution of acetone–Buffer A (2 mM Tris base, 0.2 mM CaCl_2_, 0.5 mM β-mercaptoethanol, 0.2 mM ATP at pH 8.0). A running buffer of phosphate-buffered saline (PBS, pH 7.4) was used for EMPAS measurements. Lysophosphatidic acid (LPA) was dissolved in 19:1:1 *v*/*v* of chloroform–methanol–acetic acid. The solution was divided into aliquots, evaporated, sealed under inert conditions, and stored at −18 °C. Before each measurement, one aliquot was resuspended in 1.5 mL of PBS or human serum. Ferric chloride was purchased from ACP (Montreal, QC, Canada). Hydrochloric acid was purchased from VWR (Mississauga, ON, Canada) and imidazole was purchased from Bio Basic Canada (Markham, ON, Canada). A healthy male individual voluntarily donated blood for this study; genetic or metabolic data from the sample were not collected. This study followed the ethical rules regarding research and experimentation that involve human samples.

### 2.2. Cleaning of EMPAS Quartz Crystals

EMPAS crystals (AT-cut, 13 mm diameter, 83 µm thickness, 20 MHz fundamental frequency) were purchased from Laptech Precision Inc. (Bowmanville, ON, Canada) and cleaned following previously published methods [13]. The crystals were rinsed with hot tap water, sonicated in 1% sodium dodecyl sulfate for 15 min, rinsed with hot tap water, and then rinsed with Milli-Q water (18.20 MΩ cm). Each crystal was cleaned in acidic piranha solution at 90 °C for 30 min, rinsed with Milli-Q water, and then rinsed with methanol. The crystals were sonicated in methanol for two minutes, rinsed with methanol, and then oven dried at 150 °C for 2 h. To hydrate the surface, the crystals were kept in a humidity chamber (70–80% RH) overnight at room temperature.

### 2.3. Functionalization of EMPAS Quartz Crystals

Following overnight surface hydration in a humidity chamber, EMPAS crystals were functionalized with MEG-Cl in toluene (1:1000 *v*/*v*) under inert (N_2_) and anhydrous (P_2_O_5_) conditions. After 90 min on an orbital rotator, the crystals were rinsed with toluene (×2), sonicated in toluene for two minutes, and rinsed with toluene again (×2). The MEG-Cl coated crystals were soaked individually in at least 2 mL of Milli-Q water on an orbital rotator overnight. After Milli-Q washes (×2), the crystals were exposed to 20 mM N-hydroxysuccinimide (NHS) and 50 mM 1-(3-(dimethylamino)propyl)-3-ethylcarbodiimide hydrochloride (EDC) in Milli-Q water for 35 min, rinsed with Milli-Q water (×2), then functionalized with 2 mg/mL N(α), N(α) bis-(carboxymethyl)-L-lysine disodium salt monohydrate (ab-NTA), and 2 mg/mL NiCl_2_ (Ni-NTA) for one hour. The MEG-NTA crystals were rinsed with Milli-Q water (×2) and gently dried under nitrogen. The crystals were immediately used for EMPAS measurements or stored under inert and anhydrous conditions.

### 2.4. Contact Angle Goniometry (CAG)

Static contact angle goniometry (CAG) was measured with 5 μL droplets of Milli-Q water under ambient conditions using the KSV CAM 101 contact angle goniometer (KSV Instruments Ltd., Helsinki, Finland). Measurements were conducted in triplicate and averaged. EMPAS discs functionalized with gelsolin(1-3)–actin were analyzed to compare with other functionalized layers that were previously reported in the literature.

### 2.5. Atomic Force Microscopy

Samples were scanned in tapping mode with a DI 5000 Atomic Force Microscope (Digital Instruments, Santa Barbara, CA, USA) utilizing Arrow-NCPt cantilever tips (NanoWorld, Neuchâtel, Switzerland) with a resolution of 256 × 256 points, a scan rate of 0.2–0.5 Hz, an integral gain of 0.5, a proportional gain of 1.0 and an amplitude setpoint of 0.8–1.2 V. Micrographs were analyzed using open source software (Gwyddion 2.60) to plane fit and mean level the images. 

### 2.6. EMPAS Measurements

A 1:1 ratio of gelsolin(1-3)–actin solution (50 µg/mL, 300 µL) was incubated overnight at 4 °C. The protein solution was then diluted with PBS (10 µg/mL, 1.5 mL) before injecting into the EMPAS flow-through system. LPA-spiked PBS running buffer was freshly prepared at 25 and 50 µM concentrations.

Following the standard set-up of EMPAS, measurements were performed with MEG-NTA-modified crystals at ~863 MHz with a 50 µL/min flow rate. PBS running buffer was injected until the frequency stabilized, followed by gelsolin(1-3)–actin solution at 20 µL/min for one hour. The crystal was washed with running buffer to reach frequency stabilization and then exposed to 1.5 mL of LPA-spiked running buffer, LPA-spiked human serum, or LPA-free human serum as a control. Running buffer was flowed until the frequency stabilized, allowing for the frequency shift to be determined. PBS measurements were completed in triplicate while serum measurements were conducted once as a proof-of-concept. In addition to recording frequency variations in real-time, the instrument also calculates the quality factor, which is the inverse of dissipation.

### 2.7. Nanoparticle Synthesis and Modification

Iron oxide nanoparticles (IONPs) were synthesized by the co-precipitation method as described by Safdarian and Ramezani [14]. A total of 10 mmol of FeCl_3_·6H_2_O and 5 mmol of FeCl_2_·4H_2_O were dissolved in 50 mL of 1 M HCl. The resulting solution was added dropwise to 100 mL of 2 M NaOH under a nitrogen atmosphere while refluxing at 60 °C with magnetic stirring at 800 rpm. The solution was stirred further for 1h. The black precipitate that formed was separated by an external permanent magnet, the SPHEROTM HandiMag Separator (1.287 T, Spherotech, Lake Forest IL, USA), and washed with Milli-Q water until a neutral pH was reached. The precipitate was then dried under nitrogen.

The IONPs were then modified following a procedure similar to that which De La Franier and Thompson used for silica gel [6]. Approximately 0.2 g of the IONPs was placed in a humidity chamber overnight at 80% relative humidity with a saturated aqueous solution of MgNO_3_·6H_2_O and then transferred to a test tube pre-silanized with trichloro(hexyl)silane. To this, 1 mL anhydrous toluene, 2 μL trichloro(hexyl)silane (HTS), and 1 μL MEG-Cl were added under an inert (N_2_) and anhydrous (P_2_O_5_) atmosphere in a glovebox. The test tube was capped and sealed with Parafilm™, then removed from the glovebox and placed on an orbital rotator for 1.5 h. The IONPs were rinsed with toluene, sonicated in toluene for 5 min, and sonicated in Milli-Q water for 3 min followed by a final rinse with Milli-Q water. A solution of Ni-NTA (2 mg/mL each in 2 mL water) and 1 mL pyridine were added to the IONPs. The mixture was sealed with Parafilm™ and placed on an orbital rotator overnight. Now modified with Ni-NTA, the IONPs were rinsed with Milli-Q water, dried, and then added to a solution of 1:1 gelsolin(1-3)–actin in Buffer B (2 mM Tris-Cl pH 8, 0.5 mM 2-mercaptoethanol, 0.2 mM CaCl_2_) which had been pre-incubated for 1 h. The mixture was sonicated to disperse the IONPs and then left on an orbital rotator for 1 h. The IONPs, now modified with the gelsolin(1-3)–actin complex, were washed with Milli-Q water (×3) and dried under N_2_. 

### 2.8. Nanoparticle Characterization

Samples of the IONPs were diluted in either isopropyl alcohol or ethanol and deposited onto TEM grids (Ultrathin Carbon Film on a Lacey Carbon Support Film, 400 mesh, Copper; Ted Pella Inc., Redding CA, USA). Images were obtained on a JEOL JEM 2010 High-Resolution Transmission Electron Microscope (HRTEM) (JEOL Canada Inc., Saint-Hubert, QC, Canada) at an acceleration voltage of 200 kV. Size calculations were performed using ImageJ software, version 1.54d. 

Fourier transform infrared-attenuated total internal reflection (FTIR-ATR) measurements were completed using a Perkin Elmer Spectrum Two (Perkin Elmer Inc., Greenville, TN, USA) with five scans per sample from 500 to 4000 cm^−1^. IONP samples were dried prior to measurement.

### 2.9. Nanoparticle-Chemiluminescence Measurements

A positive control assay was conducted by weighing varying amounts of IONPs and incubating them in solutions of excess imidazole (700 μM in Milli-Q water, 1 mL) for 20 min after sonication for 1 min. The excess imidazole will bind to the Ni-NTA on the nanoparticles, kicking any gelsolin–actin complexes off into the solution. The magnet was used to pull the IONPs out of solution, and the remaining liquid was assessed for chemiluminescence signal. 

Varying concentrations of 1 mL solutions of LPA in Milli-Q water were incubated with approximately 10 mg of modified IONPs for 15 min after sonication for 1 min. The magnet was used to pull the IONPs out of solution, and the remaining liquid was measured for chemiluminescence signal.

Chemiluminescence measurements were performed on 400 μL of solution by successively injecting 100 μL each of (A) 0.6% H_2_O_2_, 0.1 M HNO_3,_ and (B) 0.5 M NaOH, 4 mM cetrimonium chloride (CTAC) as suggested by Ma et al. with a total measurement time of 60 s [15]. Samples for the positive control assay were completed in replicates of three, whereas samples in the presence of LPA were completed in replicates of six.

## 3. Results and Discussion

### 3.1. Surface Characterization of EMPAS Discs

**Figure 2:**
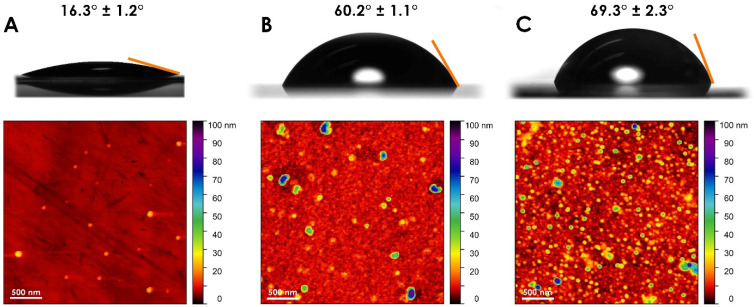
The contact angles of Milli-Q water droplets and atomic force microscopy images of (**A**) bare quartz, (**B**) gelsolin only, and (**C**) gelsolin(1-3)–actin EMPAS quartz discs.

The presence of gelsolin(1-3)–actin protein layers on the surface of EMPAS discs was confirmed by contact angle goniometry and atomic force microscopy (Figure 2). The contact angle of Milli-Q water on EMPAS quartz crystals was approximately 16.3° ± 1.2° for bare quartz, 60.2° ± 1.1° for gelsolin only, and 69.3° ± 2.3° for gelsolin(1-3)–actin functionalized surfaces. A ~9° difference occurred between gelsolin and gelsolin(1-3)–actin-modified EMPAS discs, where the protein complex was slightly more hydrophobic compared to gelsolin only. The increase in the contact angle due to the protein complex was also observed by Ahmadi et al. on stainless steel surfaces functionalized with the same linker, MEG-NTA, and gelsolin or gelsolin(1-3)–actin [16]. 

While contact angle assay is capable of distinguishing between bare and protein-modified surfaces, it does not provide evidence regarding the nature of protein coverage on the device surface. Atomic force microscopy images show that bare quartz crystals have an extremely flat surface compared to the protein-functionalized crystals. Any particulates are very small in comparison to the bound linkers and proteins. The gelsolin-functionalized surface has distinct features compared with bare quartz: an even dispersion of globules is distributed across the surface. Finally, the gelsolin(1-3)–actin-functionalized surface has the most height variation with respect to surface topography. The change in surface topography in the region of ~0–20 nm is likely due to the presence of gelsolin(1-3)–actin complexes as gelsolin and actin have similar diameters of ~5 nm [17,18], with the complex having a dimension of ~10 nm. The greater variability in surface topography could also be due to small oligomers of actin, which have a diameter of ~8–9 nm [19] with varying lengths. The gelsolin(1-3)–actin image is similar in particle size to that evident in a study by He et al., where actin appears to have lengths in the range of 5–25 nm [20]. The AFM images indicate a distinct qualitative difference between the protein-modified surfaces compared to unfunctionalized quartz.

### 3.2. Iron Oxide Nanoparticle Characterization

#### 3.2.1. FTIR-ATR of Bare and Modified Iron Oxide Nanoparticles

**Figure 3:**
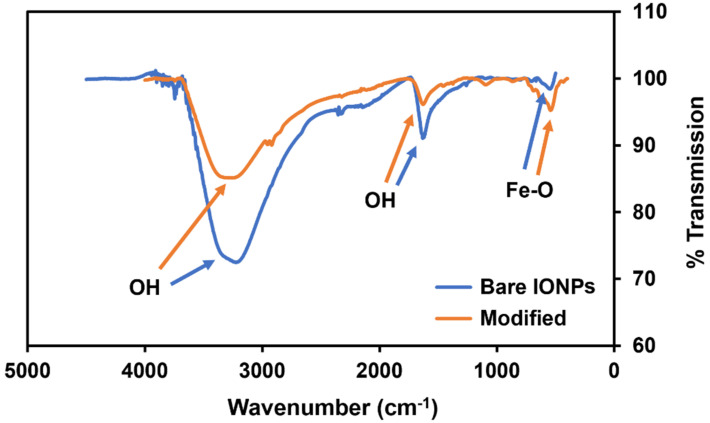
FTIR-ATR spectra of bare iron oxide nanoparticles, and iron oxide nanoparticles coated with HTS, MEG-Cl, Ni-NTA, and gelsolin(1-3)–actin–acridinium (see the legend).

The FTIR-ATR results for the bare and modified IONPs in Figure 3 show the expected Fe-O stretching peak around 500 cm^−1^. Both samples also show strong peaks from the presence of the hydroxyl groups on the nanoparticle surface, as demonstrated by the very deep and broad peaks around 3500 cm^−1^. The broad O-H band in the bare IONPs shifts closer to 3700 cm^−1^ in the fully modified sample, indicating the addition of expected N-H stretching bands from the proteins added to the surface. The reduction in the intensity of this band in the modified IONPs also indicates the change of surface hydroxyls as they form bonds with the MEG-Cl linker molecules. While peaks around 1620 cm^−1^ are often attributed to C=C stretching, in iron oxide nanoparticles they tend to represent bending vibrations in OH groups [21]. This band has previously been shown to decrease in intensity in core-shell structures compared with bare structures, which is also evidenced here [21]. More bands may be expected to be visible in the modified IONPs, but any signal from the modification layers is dwarfed by the signal from the IONP core, as the size of the core is significantly larger than the size of the modified layers. These bands demonstrate successful modification of the IONPs.

#### 3.2.2. Transmission Electron Microscopy of Bare and Modified Iron Oxide Nanoparticles

**Figure 4:**
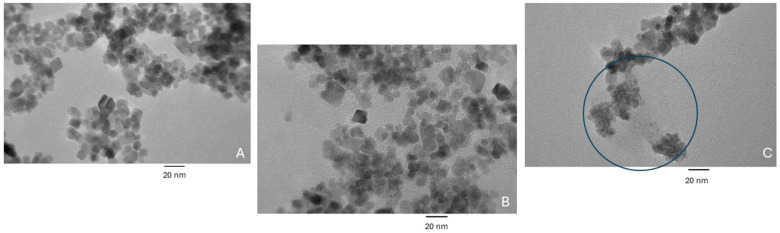
TEM images of (**A**) bare iron oxide nanoparticles, (**B**) iron oxide nanoparticles coated with HTS, MEG-Cl, and Ni-NTA, and (**C**) iron oxide nanoparticles coated with HTS, MEG-Cl, Ni-NTA, and gelsolin(1-3)–actin–acridinium. The circled area in (**C**) depicts protein aggregates.

Figure 4 shows TEM images of the (A) bare IONPs, (B) IONPs after modification with the linker (MEG-Cl), the spacer (HTS), and Ni-NTA, and (C) IONPs after full modification with the above and the gelsolin(1-3)–actin–acridinium dye complex. The images show an average particle diameter of 14.42 ± 2.57 nm and a tendency of the particles to form aggregates. There is no significant difference visible between the bare IONPs and the first modification step; however, in the fully modified IONPs, aggregates of smaller particles are seen. These smaller aggregates are attributed to the protein that has been unintentionally removed from the sample due to the preparation in ethanol, which interfered with the linker chemistry. While the removal of the protein from the surface was unintentional, the presence of these aggregates confirms that the protein was in fact bound to the surface of the IONPs before incubation with ethanol which reacted with the MEG-Cl linker. In further imaging with different solvents, these small protein aggregates were not visible, indicating that they remained bound to the nanoparticle surface. Due to the tendency of the particles to aggregate in various solvents, the IONPs were dried and weighed out for use in further experiments.

### 3.3. Testing the Gelsolin(1-3)–Actin Protein Sensors for Lysophosphatidic Acid Quantification

#### 3.3.1. Acoustic Wave Analysis with EMPAS

Frequency shifts are only proportional to changes in mass if the adlayer on the EMPAS disc has a weak or negligible contribution from viscosity. In other words, the layer must be rigid rather than viscoelastic. Determining the viscoelasticity or rigidity of an adlayer has been studied for thickness-shear mode (TSM) sensors, where a layer is considered stiff if the ratio of the dissipation shift over frequency shift is less than the inverse of the fundamental frequency [22]:

$$\frac{{∆D}_{n}}{{∆f}_{n}}\ll \frac{1}{f_{0}}$$

where ${∆D}_{n}$ is the change in dissipation at harmonic *n*, ${∆f}_{n}$ is the change in frequency at the same harmonic *n*, and $f_{0}$ is the fundamental frequency. In our case, the fundamental frequency of the EMPAS disc is around 20 MHz, and therefore 1/(2 × 10^7^ Hz) = 5 × 10^−8^ Hz^−1^.

Another criterion for determining the stiffness of an adlayer is by comparing the ratio to values; the adlayer is considered rigid if ${∆D}_{n}$/${∆f}_{n}$ < 10^−8^ Hz^−1^ [23], while a viscoelastic layer occurs if ${∆D}_{n}$/${∆f}_{n}$ > 4 × 10^−7^ Hz^−1^ [24].

As TSM and EMPAS are both based on bulk acoustic waves, we can apply this calculation to determine the rigidity of the gelsolin(1-3)–actin layer. If the adlayer is more rigid than purely viscoelastic, the frequency shift is approximately proportional to mass changes on the surface [22,23,24]. The frequency shift of gelsolin(1-3)–actin binding to the crystal surface is 3.3 kHz, while the dissipation shift is 4.9 × 10^−6^. Therefore, the ratio of dissipation shift over frequency shift is as follows:

4.9 × 10^−6^/3.3 × 10^3^ Hz ≈ 1.5 × 10^−9^ Hz^−1^

Since 1.5 × 10^−9^ Hz^−1^ < 5 × 10^−8^ Hz^−1^, therefore ${∆D}_{n}$/${∆f}_{n}$ ≪ 1/$f_{0}$ and ${∆D}_{n}$/${∆f}_{n}$ < 10^−8^ Hz^−1^ apply to our system, indicating that the gelsolin(1-3)–actin layer is not particularly viscoelastic. As a result, the removal of actin mass from the adlayer can be approximately correlated with changes in frequency. The following calculations of mass adsorbed and removed must be considered approximations as there is some minor error due to the minimal viscoelastic properties of the biomaterials of the assay.

**Figure 5:**
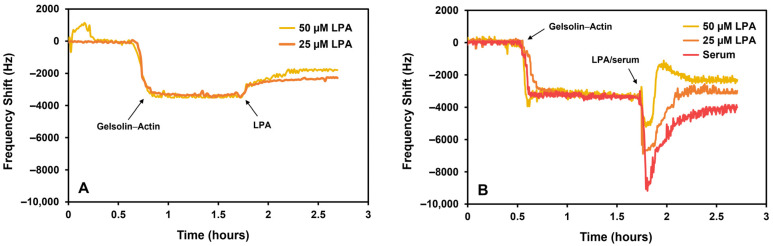
EMPAS response to LPA in (**A**) phosphate-buffered saline and (**B**) serum at the 43rd harmonic.

The resonance frequency increased following incubation with 25 μM or 50 μM LPA in PBS and human serum (Figure 5). The increase in frequency corresponds to the removal of actin protein from the mass-sensitive surface as a result of LPA dissociating the protein complex. Since actin and gelsolin(1-3) have similar molecular weights (41.78 kDa [25] and 39 kDa [26], respectively), the removal of actin can be a maximum of half the frequency shift that corresponds with gelsolin(1-3)–actin binding. The initial frequency drop for serum or LPA-spiked serum in Figure 5B can be the result of the sensor responding to a change in the density of the flowing liquid (from buffer to serum).

The binding of gelsolin(1-3)–actin to the surface of the resonating device caused a decrease in frequency by 3.3 ± 0.1 kHz. A previous work [27] investigated the specific adsorption of gelsolin(1-3) onto MEG-NTA-coated crystals, which produced a frequency shift of about 4 kHz, using the 53rd harmonic (around 1064 MHz). To compare the data, the normalization of the different harmonics is required and is achieved by dividing the frequency shifts by their corresponding harmonic number:

4.1 kHz/53 ≈ 77 Hz 3.3 kHz/43 ≈ 77 Hz

The previous work did not involve the actin protein, only gelsolin. However, as our present study obtained the same trend in frequency variation (77 Hz) during protein binding, this indicates that gelsolin(1-3)–actin binds to the surface horizontally, forming a monolayer. If the protein complex would immobilize vertically and form a bilayer, the frequency shift would approximately double as the mass would double.

The frequency shift in PBS after 25 μM LPA was around 1.1 kHz, corresponding to ~34.2% of protein removed. For 50 μM LPA in PBS, the frequency shift was around 1.7 kHz, which corresponds to ~48.2% of protein removed. The sensor is nearly saturated when exposed to 50 μM LPA, as ~50% of the protein must be removed. Since mass is directly proportional to frequency variation in this system, we can relate the frequency shift to calculate the percentage of mass removal (M%):

$$M\%\approx \frac{{\Delta f}_{LPA}}{{\Delta f}_{GA}}\times 100$$

where ${\Delta f}_{LPA}$ and ${\Delta f}_{GA}$ are the frequency shifts associated with LPA and gelsolin(1-3)–actin, respectively. 

As expected, serum adsorption is minimal due to the sensor’s antifouling properties, causing a frequency shift of approximately 760 Hz (normalized to 18 Hz). In the previous work [27], the fouling of serum was 2 kHz (normalized to 38 Hz). Serum fouling in our current work is reduced due to the presence of gelsolin(1-3)–actin, which prevents further non-specific adsorption.

In serum, 50 μM LPA caused an increase in frequency by ~710 Hz, while 25 μM LPA shifted the frequency by ~360 Hz. To calculate the mass of protein removed, the contribution of serum adsorption must be considered (~760 Hz). The previous equation used for PBS measurements is modified for the serum (assuming that serum adsorption is consistent):

$$M\%\approx \frac{{\Delta f}_{LPA}+{\Delta f}_{SERUM}}{{\Delta f}_{GA}}\times 100$$

where ${\Delta f}_{SERUM}$ is the frequency shift (760 Hz) associated with serum adsorption. In serum, 25 μM LPA removed ~31.7% of protein, while 50 μM LPA removed ~45.6%. The similar amount of protein removed in both PBS and serum indicates that the sensor is capable of effective quantification of LPA in complex biological samples. The slight decrease in protein removed in serum compared to the buffer is due to the serum environment influencing LPA’s activity.

**Figure 6:**
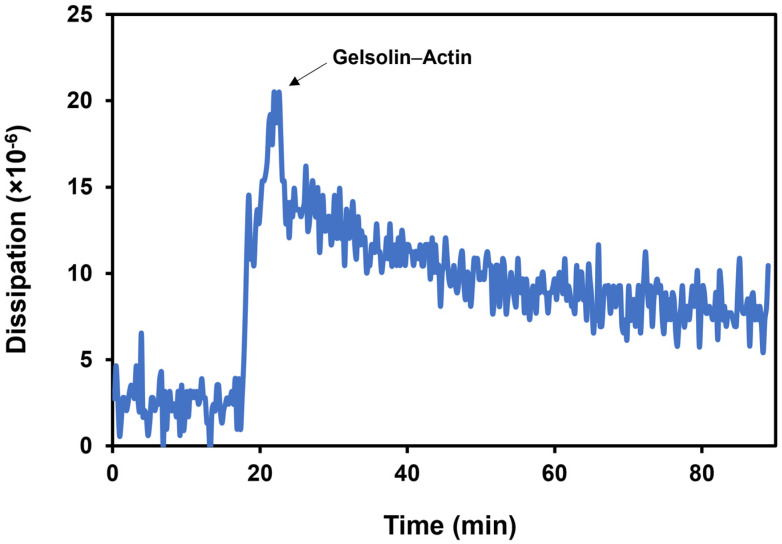
The dissipation shift upon gelsolin(1-3)–actin binding in phosphate-buffered saline.

The dissipation of the acoustic energy increases upon gelsolin(1-3)–actin binding since biomolecules such as proteins generally have viscoelastic properties (Figure 6) [28]. An average dissipation shift of (4.9 ± 0.7) × 10^−6^ was obtained following the binding of the dual-protein system. Since dissipation occurs, the aforementioned percentages of mass removed are approximations due to the minimal contribution from the viscoelasticity of the protein layer.

#### 3.3.2. Iron Oxide Nanoparticles and Chemiluminescence 

**Figure 7:**
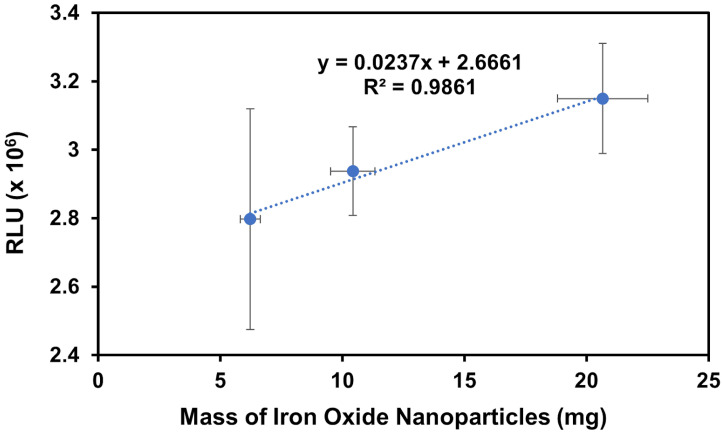
A plot of RLU vs. mass of the modified IONPs using high imidazole concentrations to compete with gelsolin binding to Ni-NTA moieties as a positive control experiment to confirm the successful modification. Note that 0 μM LPA was tested but omitted from the figure for clarity, as it gave a much lower signal of 0.1 × 10^6^ RLU.

The positive control results in Figure 7 display an upward trend in chemiluminescence in the relative luminescence unit (RLU) with increasing amounts of IONPs, as expected. Since the gelsolin(1-3)–actin–dye complex is attached to the IONPs via a histidine tag, imidazole competes with this interaction, removing protein–dye complexes from the IONPs and depositing it into the solution which indirectly mimics what should happen in the presence of LPA. Thus, with higher masses of nanoparticles, more dye is present in the solution, resulting in higher chemiluminescence signals. The R^2^ value of 0.9861 is promising, though the vertical error bars are larger than desired due to the difficulty in reproducing the same masses for each sample. This is discussed further below. The presence of any signal, but especially an increasing signal, also demonstrates that the IONPs were successfully modified with the gelsolin(1-3)–actin–acridinium complex.

**Figure 8:**
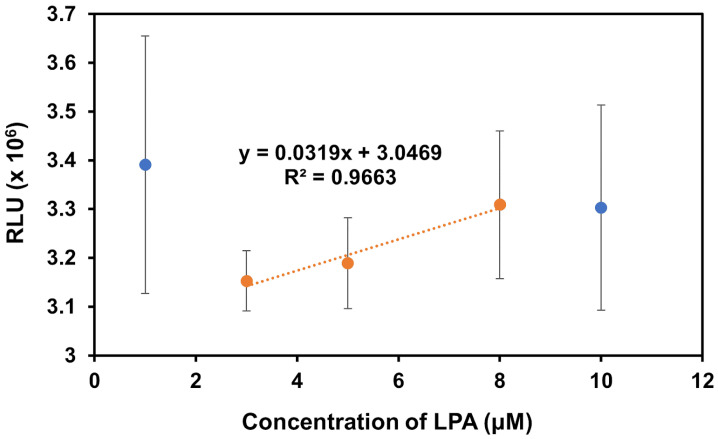
The chemiluminescence response of the modified IONPs in the presence of varying concentrations of LPA in Milli-Q water. The trendline and associated equation were obtained from only the orange data points. The blue data points represent the 1 μM and 10 μM solutions which were measured but excluded from the trendline due to their high levels of variability.

In the presence of LPA, there is a clear upward trend in signal with increasing LPA concentration, as expected, except for the solutions of 1 μM and 10 μM (Figure 8). The 1 μM solution was removed from the trendline due to the high variability in this sample, likely drawing the signal out of the expected correlation pattern. The 10 μM sample was removed from the trendline as previous experiments with the acridinium-NHS-ester dye indicated saturation was reached at this concentration, which aligns with the observed high variability in measurements of this sample. In the absence of these outlier points, we see a strong correlation with R^2^ = 0.9663, indicating the preliminary success in the ability of the biosensor to detect changes in LPA concentration. Further work will prioritize optimizing the assay to reduce sample variability and detect lower concentrations.

**Figure 9:**
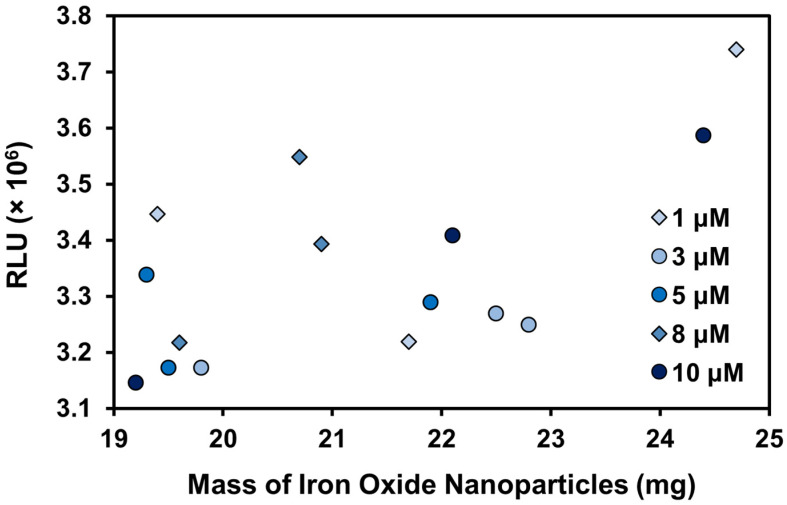
The detected RLU correlated with the mass of IONPs in each sample. Each series represents the concentration of LPA that the particular mass of IONPs was incubated with.

To assess if the varying mass of IONPs in each sample contributed to the large variability in the chemiluminescence signal, the same data in Figure 8 was plotted in Figure 9 as the signal generated by the mass of IONPs in a specific sample. The data have been separated into series based on the concentration of LPA the IONPs were immersed into. If the mass of IONPs did not contribute to the observed signal, there would be a clear gradient in colour visible in Figure 9 with the highest signals obtained by the highest concentrations of LPA (represented by the darkest colour), and the lowest signals by the lowest concentrations (represented by the lightest colour). There would be no apparent correlation between the signal and the mass of IONPs. The data in Figure 9 clearly show that at least some of the variability in the signal can be attributed to the increased mass of IONPs in certain samples. Across most samples from the same concentrations, higher masses sometimes give higher signals. For example, the sample with 24.7 mg of IONPs, which resulted in the highest signal in Figure 9, was the sample incubated with 1 μM LPA.

The mass of IONPs should not affect the signal generated if the correct amount of particles (and therefore dye) were used. The variable chemiluminescence signal may be that all dye is removed from the IONPs during sample preparation because there is not enough dye present to react with the specified concentrations of LPA. Further experiments with higher masses of IONPs need to be conducted. Additionally, if the mass continues to contribute to the variation in signals, drying and weighing the particles is not a suitable way forward; however, this method of preparation was originally chosen due to the tendency of the particles to aggregate as evidenced by the TEM images in Figure 4. Thus, it is necessary to further assess the modification procedure to see if solution dispersion can be improved and if particles can be measured by volume instead of mass to reduce variability. It is likely that the surface chemistry, which must be performed under a nitrogen atmosphere and was chosen for its antifouling properties, also affects the solubility of the IONPs, the strength of the bonds between the protein–dye complex and the IONPs, and therefore the performance of the assay. While antifouling is important for large, flat surface areas, questions remain as to whether antifouling properties are necessary for particles of this size scale. We plan to further investigate the necessity of antifouling for this specific assay and look to improve the solution dispersion of the IONPs.

## 4. Conclusions

The protein complex gelsolin–actin was applied as a probe for detecting lysophosphatidic acid (LPA) since LPA binding causes the decoupling of the complex. The electromagnetic piezoelectric acoustic sensor (EMPAS) and chemiluminescence sensor based on iron oxide nanoparticles (IONPs) were designed and characterized to develop prospective LPA assays. Preliminary proof-of-concept results in Milli-Q water, buffer, and/or human serum are promising, demonstrating the potential of these biosensors to be developed for rapid and quantitative sensing of the ovarian cancer biomarker. 

For the EMPAS crystals, contact angle goniometry confirmed functionalization based on hydrophobicity while atomic force microscopy visualized the qualitative difference between bare quartz and protein-functionalized surfaces. Variability in surface morphology increased for gelsolin–actin surfaces compared to gelsolin only, indicating that our procedures lead to successful functionalization.

Importantly, the complex matrix of unprocessed serum has a negligible effect on LPA’s activity as high concentrations of LPA caused an increase in frequency, indicating actin mass removal. The results demonstrate a proof of concept for the acoustic LPA assay as the same trend was observed in both buffer and serum. Furthermore, fouling of serum following adsorption of gelsolin–actin was further reduced compared to our previous work [27]. Compared to previous results with gelsolin binding [27], the frequency shift due to gelsolin–actin immobilization indicates that the complex binds horizontally, as a monolayer. 

Successful modification of the IONPs with the gelsolin–actin–dye complex was confirmed qualitatively with FTIR spectra and TEM imaging. The FTIR showed the change in surface functional groups throughout the modification procedure, and TEM imaging indicated the presence of the protein on the IONPs by showing that protein was removed and aggregated when the IONPs were dissolved in ethanol. 

Assays conducted with the IONPs both in the presence of imidazole (as a positive control) and LPA are a proof of concept that the protein–dye complex was successfully attached to the IONPs, and the sensor can detect LPA indirectly through the displacement of actin–dye from gelsolin on the IONP surface. The responses show a strong correlation between signal generation and concentration. Currently, the variability in results is high, but this is likely due to the variability in the mass of IONPs used as it is difficult to consistently weigh out these small masses. Future work will attempt to modify the surface chemistry of the IONPs to reduce aggregation and allow for the measurement of particles using volume instead of mass, which should reduce variability significantly. 

Additional studies can include (1) testing various antifouling linkers to compare the antifouling ability in serum and measuring at higher acoustic harmonics to study the sensitivity for the EMPAS assay, and (2) altering the amount of IONPs in samples, improving the solubility of the IONPs, as well as an investigation of whether the antifouling properties of MEG-Cl are necessary on the size scale of nanoparticles for the chemiluminescence assay. Future work will determine if the acoustic and chemiluminescence sensors can detect LPA with a low limit of detection in serum that can distinguish between good health and ovarian cancer. 

## Data Availability

Data are contained within the article.
